# Structural Safety Analysis Based on Seismic Service Conditions for Butterfly Valves in a Nuclear Power Plant

**DOI:** 10.1155/2014/743470

**Published:** 2014-05-11

**Authors:** Sang-Uk Han, Dae-Gyun Ahn, Myeong-Gon Lee, Kwon-Hee Lee, Seung-Ho Han

**Affiliations:** Department of Mechanical Engineering, Dong-A University, Busan 604-714, Republic of Korea

## Abstract

The structural integrity of valves that are used to control cooling waters in the primary coolant loop that prevents boiling within the reactor in a nuclear power plant must be capable of withstanding earthquakes or other dangerous situations. In this study, numerical analyses using a finite element method, that is, static and dynamic analyses according to the rigid or flexible characteristics of the dynamic properties of a 200A butterfly valve, were performed according to the KEPIC MFA. An experimental vibration test was also carried out in order to verify the results from the modal analysis, in which a validated finite element model was obtained via a model-updating method that considers changes in the *in situ* experimental data. By using a validated finite element model, the equivalent static load under SSE conditions stipulated by the KEPIC MFA gave a stress of 135 MPa that occurred at the connections of the stem and body. A larger stress of 183 MPa was induced when we used a CQC method with a design response spectrum that uses 2% damping ratio. These values were lower than the allowable strength of the materials used for manufacturing the butterfly valve, and, therefore, its structural safety met the KEPIC MFA requirements.

## 1. Introduction 

The availability factor of nuclear power plants has been significantly improved worldwide, whereby nuclear power is becoming more economically competitive with fossil fuels for base-load electricity generation in many countries [1]. Nuclear power accounted for nearly 35% of domestic production electricity in 2012 and is gradually increasing in dependency [2]. Structural integrity includes valves that can withstand earthquakes and other dangerous situations, because they are used to control cooling waters in the primary coolant loop to prevent boiling within the reactor in a nuclear power plant. An accident due to the leakage of radioactive matters, however, can inflict catastrophic damage on the environment nearby. Therefore, with the enhanced awareness of the potential of an earthquake to cause such damage, qualifying the valves has now become standard practice, that is, establishing their ability to withstand a seismic load without damage. Strict safety guidelines should be carried out as defined by the KEPIC MFA [3], which indicates the verification of seismic adequacy with prescribed safety rates for structures and equipment. The verification of the seismic adequacy consists of environmental qualifications for handling the effects of heat- and radiation-induced degradation and of seismic qualifications that should be carried out either by numerical analysis or by experimental tests using a shake-table or by comparison with past experiences. The seismic qualification for the use of shake-table testing is normally very exorbitant and these facilities may not be available in many places and can show only single isolated structures or equipment without simulating structural connections to the secondary component, which may change the dynamic behavior when compared with the actual as-installed structures [4, 5]. Another venerable method for seismic qualification is purely analytical and uses a finite element method. The reliability of this method totally depends on the finite element model, which generally cannot produce the dynamic behavior of as-installed structures even for structurally simple components. Thus, finite element models are usually verified via an experimental modal test. The test results are assumed to be correct and the finite element model is tuned to closely correlate with the experimental test results.* In situ* modal test data obtained from the modal tests conducted on the as-installed structure were used directly for the seismic response estimation in order to overcome the limitations when using a finite element model for seismic analysis [6, 7]. However, these methods may not always be practical for many of the structural components in a nuclear power plant due to the difficulties of conducting* in situ* modal tests.

In the present study, the numerical analyses using finite element methods, that is, static and dynamic analyses according to the rigid or flexible characteristics of dynamic properties for a 200A butterfly valve in a nuclear power plant, were performed according to the dictates of the KEPIC MFA [3]. An experimental vibration test was carried out in order to verify the results from the modal analysis, whereby a validated finite element model was obtained via model updating that considered the changes in* in situ* experimental data. By using the validated finite element model, structural safety analysis under seismic service conditions was carried out.

## 2. Seismic Qualification

### 2.1. Butterfly Valve

**Figure 1:**
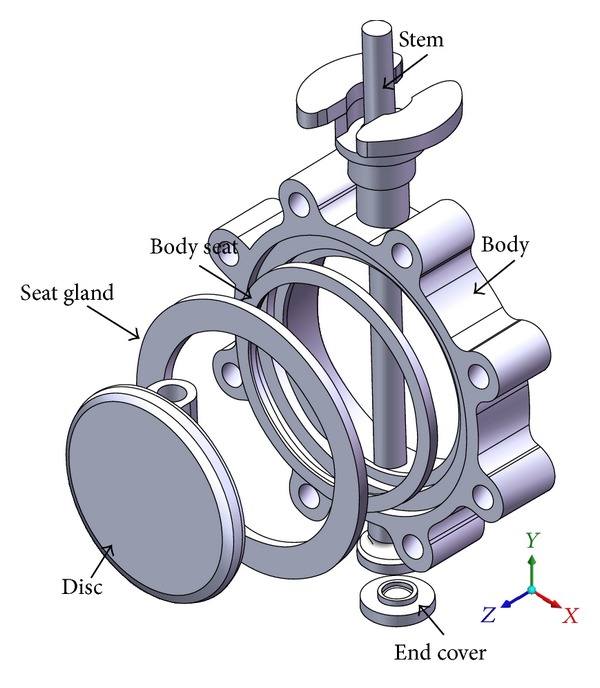
Configuration of a 200A butterfly valve.

Figure 1 shows the configuration of the butterfly valve, which is used in a nuclear power plant to control cooling waters in the primary coolant loop preventing boiling within the reactor. Since a radiation leak would have a disastrous effect on the environment, the butterfly valve must not leak and must endure earthquakes and other dangerous situations. The butterfly valve shown in Figure 1 has a 200 mm inner diameter and consists of 6 parts: stem, body, body seat, seat gland, disk, and end cover. These parts are made up of the following materials: Gr. WCB, CF8, T316, T316, A564-630, and T304, respectively.

### 2.2. Seismic Qualification Process

The seismic qualification of the butterfly valve should demonstrate that the valve has the ability to perform its safety functions during and after being subjected to the forces resulting from a safe shutdown earthquake (SSE) event. The KEPIC MFA [3] describes the approach methods for seismic qualification, which are grouped into 4 general categories: to predict the equipment's performance by numerical analysis, to test the equipment under simulated seismic conditions, to qualify the equipment by a combination of experimental tests and numerical analyses, and to qualify the equipment through the use of experience data. Each of the categories may be adequate to verify the ability of the equipment to meet the seismic qualification requirements. Among these 4 categories, this study focused on predicting the equipment's performance via numerical analysis.

**Figure 2:**
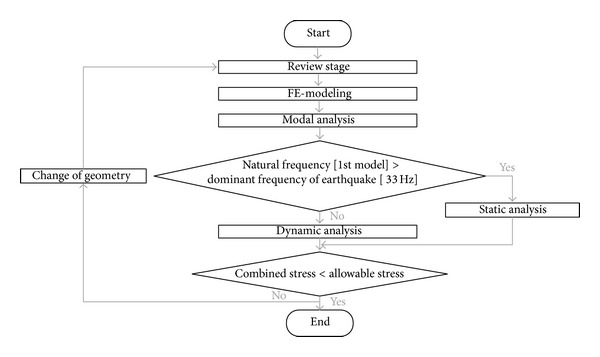
Flow chart of performance assessment using numerical analysis.

The methods used for numerical analysis include static and dynamic analysis depending on the structure of the equipment and dynamic properties such as the complexity of the equipment and whether the equipment is rigid or flexible [8].  Figure 2 shows the flow chart of a performance assessment based on a seismic qualification using numerical analysis according to the KEPIC MFA [3]. The review stage in the first step takes into account the complexity of the butterfly valve and the adequacy of analytical techniques to properly predict its safe operation during seismic excitation. The butterfly valve should be modeled such that its mass distribution and stiffness characteristics will be adequately represented when using the finite element method. This finite element model can be used to perform a modal analysis in order to determine the rigidity or flexibility. When the natural frequency at the 1st mode calculated by the modal analysis is higher than a cut-off frequency of 33 Hz, that is, the dominant frequency of an earthquake, the butterfly valve is considered sufficiently rigid and may be analyzed statically. But if it is not higher than 33 Hz, a dynamic analysis should be performed because the butterfly valve is considered to be flexible and poses a risk of resonance in the dominant frequency range of an earthquake. In this step, the butterfly valve can be analyzed via response spectrum analysis, in which the responses of stress obtained from each modal response are combined to consider all significant modes. Finally, the structural safety is estimated by comparing the combined stress with the allowable stress of the materials in use.

#### 2.2.1. Static Analysis

Static analysis, also known as equivalent static force analysis, is a method that enables calculation of the stresses in each part of the structure to be recreated by a static force, that is, the equivalent of an earthquake. Although static analysis often underestimates structural safety by comparison with dynamic analysis, this simple process prevents the need to perform time-consuming computations. The acceleration responses that are required in order to estimate structural safety during analysis must be determined only according to the maximum peak of the response spectrum using a conservative damping value. The seismic qualification for unit of equipment devices or structural systems can be achieved only by static analysis, because the effect of resonance does not have to be considered. The governing equation of static analysis can be presented as follows:

(1) $$\begin{array}{c} \left[K\right]\left\{U\right\}=\left\{F\right\}, \end{array}$$

where [*K*], {*U*}, and {*F*} are designated as the stiffness matrix, the nodal displacement vector, and the external force vector, respectively, caused by the dead load or gross weight. The nodal displacement vector {*U*} was calculated using the finite element method, which then allowed for the stress distribution at nodal points.

#### 2.2.2. Dynamic Analysis

For flexible equipment, where the natural frequency is lower than the dominant frequency of an earthquake, that is, 33 Hz, as shown in Figure 1, dynamic analysis should be carried out based on either the response spectrum method or the time history method. The response spectrum method based on structural dynamics enables the approximate estimation of the dynamic performances of the equipment such as the maximum responses of displacement and stress. The dynamic performances are determined by combining each modal response, which includes all significant modes. This method is commonly used for the dynamic analyses of seismic qualifications. Meanwhile, the time history method can be used to evaluate the time history of dynamic responses due to an earthquake, which displays earthquake-induced motion as a function of time, usually in terms of acceleration. Although the time history method provides relatively accurate dynamic responses, a time-consuming computational work and a complicated procedure are required due to the consideration of a large number of degrees of freedom and to the detailed data from earthquake-induced motion [9, 10]. In the present study, dynamic analysis was conducted using the response spectrum method. The dynamic performances of the equipment can be presented as follows:

(2) $$\begin{array}{c} \left[M\right]\left\{\ddot{U}\right\}+\left[C\right]\left\{\dot{U}\right\}+\left[K\right]\left\{U\right\}=\left[P\left(t\right)\right], \end{array}$$

where [*M*], [*C*], $\left\{\ddot{U}\right\}$, $\left\{\dot{U}\right\}$, and [*P*(*t*)] are the mass matrix, the damping matrix, the nodal acceleration vector, the nodal velocity vector, and the applied dynamic load vector, respectively. In the analysis procedure, the nodal velocity vector of the finite element analysis is obtained first, and then the nodal displacement and stress can be calculated. The modal analysis must be carried out before application of the response spectrum analysis, because [*P*(*t*)] is designated as the set of load values induced from the modal responses. After the modal analysis, a response spectrum analysis should be carried out, whereby the responses of stress obtained from each modal response combine all significant modes. There are two rational ways to combine responses from the response spectrum: the SRSS (square root of sum of square) method and the CQC (complete quadratic combination) method [11, 12]. In the SRSS method, the squares of a specific response are summed, and the square root of this sum takes the combined effect into account. The SRSS provides relatively conservative results, except where closely spaced modes apply. In the case of closely spaced modes, the combined response values are often underestimated. Meanwhile, the CQC method combines responses based on the use of cross-modal coefficients, which reflect the duration and frequency content of the seismic events as well as the modal frequencies and damping ratio of the equipment. The present study applied the CQC method. The total mode response, *R*_*a*_, obtained by the CQC method can be written as follows:

(3) $$\begin{array}{c} R_{a}={\left[\overset{N}{\underset{i=1}{\sum }}\;\overset{N}{\underset{j=1}{\sum }}k\varepsilon_{ij}R_{i}R_{j}\right]}^{1/2}, \end{array}$$

where *k* is 1 when *i* = *j* is valid and is 2 when *i* = *j* is invalid; *R*_*i*_ and *R*_*j*_ represent the mode responses at the *i*th and *j*th modes, respectively. *ε*_*ij*_ designates the cross-modal coefficient presenting the correlation between the *i*th and *j*th modes, which can be presented as follows:

(4) $$\begin{array}{c} \varepsilon_{ij}=\frac{8{\left(\xi_{i}\xi_{j}\right)}^{1/2}\left(\xi_{i}+r\xi_{j}\right)r^{3/2}}{{\left(1-r^{2}\right)}^{2}+4\xi_{i}\xi_{j}r\left(1+r^{2}\right)+4\left(\xi_{i}^{2}+\xi_{i}^{2}\right)r^{2}}, \end{array}$$

where *r* and *ξ* are the ratio of natural frequencies and modal damping, respectively.

## 3. Modal Analysis

### 3.1. Modal Analysis Using the Finite Element Method

**Figure 3:**
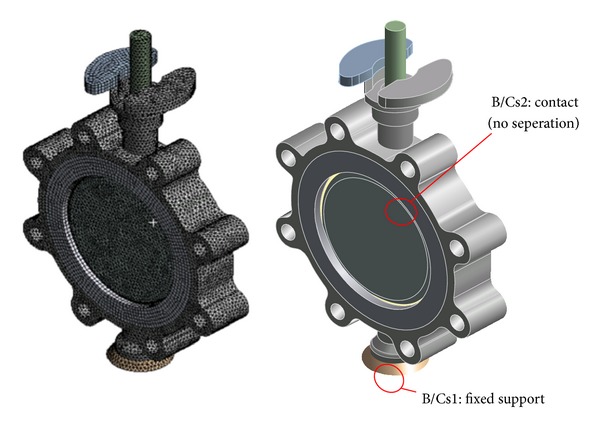
Finite element model and boundary conditions for modal analysis based on static analysis.

**Figure 4:**
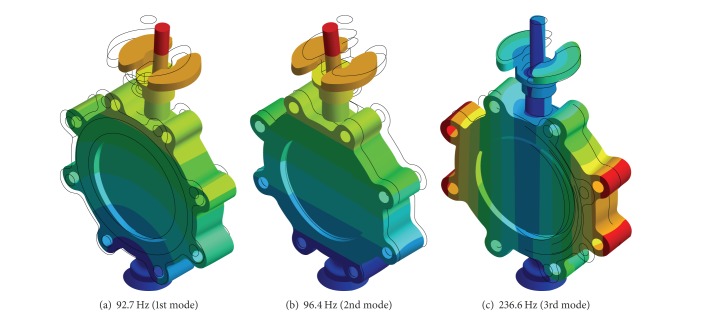
Frequencies at each mode obtained by modal analysis.

The modal analysis for the butterfly valve was performed using the finite element method via the commercial software, ANSYS Workbench [13].  Figure 3 shows the finite element model constructed using the preprocessor option provided in ANSYS Workbench with the boundary conditions applied to the modal analysis. The contact conditions between each part of the finite element model were implemented using special elements, such as CONTA 174 and TARGE 170, and the no-separation contact boundary condition, where a sliding, but not nonlinear, motion is permitted at the contact surfaces between the disc and body sheet. Furthermore, to investigate the effect of the contact conditions on the frequencies occurring at each mode, other boundary conditions show that the contact surfaces are bonded.  Figure 4 shows the natural frequencies obtained by the modal analysis under the no-separation contact boundary conditions, that is, 92.7, 96.4, and 236.6 Hz at the 1st, 2nd, and 3rd modes, respectively. In the case of the bonded-surface boundary conditions, the modal analysis provided natural frequencies of 94.4, 96.3, and 240.4 Hz for each mode, which was a less than 2% error compared with that of the no-separation contact boundary conditions. These results showed that the natural frequencies of the butterfly valve were higher than 33 Hz, which means that the butterfly valve can be considered sufficiently rigid and could be analyzed statically according to the process of seismic qualification, as shown in Figure 2.

### 3.2. Modification of the Finite Element Model

The reliability of the modal analysis totally depends on the finite element model. If the mass distribution and boundary conditions of the finite element model cannot be considered equivalent to the as-installed conditions, the dynamic behavior obtained from the finite element model shows a significant difference compared with the as-installed version. Therefore, the finite element model should be verified using the data from the experimental modal test, in which the test results are assumed to be correct, and the finite element model is tuned to closely correlate with the test results. In the present study, we performed a model-updating method that considered changes in the* in situ* experimental modal test data, and a validated finite element model was obtained.

**Figure 5:**
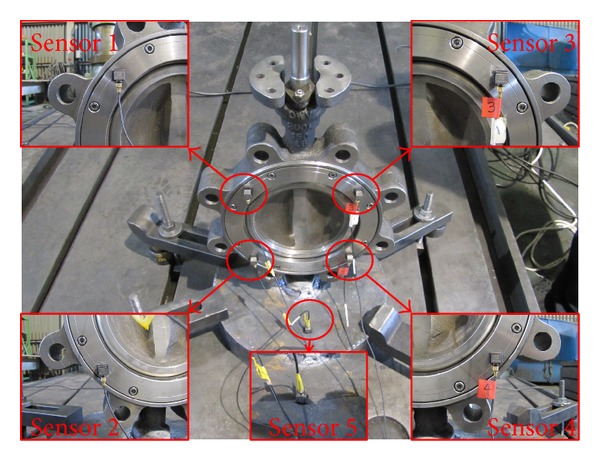
Accelerometers attached on the butterfly valve for the experimental modal test.

**Figure 6:**
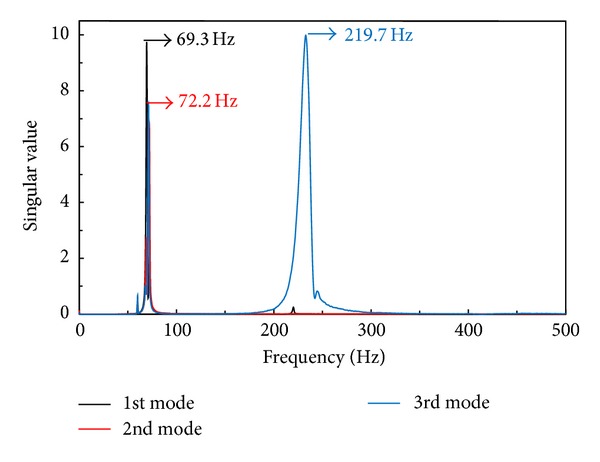
Singular values related to the frequencies for each mode.

An experimental modal test of the butterfly valve was performed. The end cover of the butterfly valve was welded to a steel plate on the reaction floor. The butterfly valve was instrumented with 5 accelerometers from the B&K Co. [14] with a capacity of 3,000 G, as shown in Figure 5. The location of the accelerometers was selected according to the numerical results from the modal analysis, where 4 accelerometers measured the acceleration at each mode, and the other accelerometer was attached to the steel plate on the reaction floor to compensate for the relative movement of the butterfly valve. Data acquisition was accomplished using NEXUX software from the B&K Co. Singular values of acceleration data occurred by the stroke of an impact hammer were converted into natural frequencies at each mode.  Figure 6 shows natural frequencies of 69.3, 72.2, and 219.7 Hz at the 1st, 2nd, and 3rd modes, respectively, and these are listed in Table 1. As the table shows, the experimental values deviated significantly more than 25% from the computed frequencies using the initial finite element model. This might have been caused by a poor reflection of the initial finite element model for the butterfly valve such as simplified and idealized assumptions made while constructing the finite element model. For the numerical modal analysis, the butterfly valve was assumed to be rigidly fixed at the steel plate. However, during the experimental modal test, the butterfly valve had either a small degree of rotation or a small deflection at the fixed point. The initial finite element model had to be modified so that it would project confidence for further analysis. To modify the initial finite element model, a trial and error method was used, which is a common part of the model-updating method [15]. Based on the physical understanding of the installation of the butterfly valve, the boundary stiffness at the rigidly fixed steel plate was adjusted, which brought the finite element model predictions close to the experimental results. As shown in Table 1, the computed frequencies from the updated finite element model are almost identical to the measured ones.

## 4. Results of the Structural Safety Analysis

### 4.1. Static Analysis

**Figure 7:**
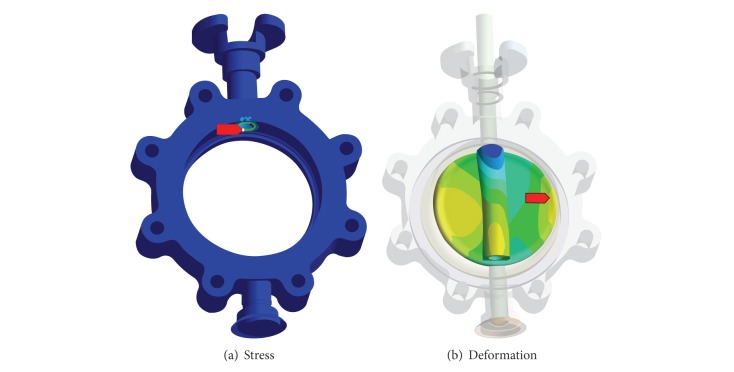
Results of structural analysis under reverse pressure according to Grade D of the KEPIC MFA.

The structural safety analysis of the butterfly valve was carried out by the seismic qualification based on the static analysis using the validated finite element model, in which the butterfly valve can be considered rigid due to all natural frequencies of higher than 33 Hz, as calculated by the modal analysis. The stresses applied in the butterfly valve were calculated under a combined load, that is, an equivalent static force, defined as Grade D by the KEPIC MFA. In the case of Grade D, the combined load, which was subjected at the center of gravity in the butterfly valve, accounted for dead weight, operation load, and SSE load. The operation load occurred under normal and reverse pressures with respect to flow direction in the butterfly valve, and the SSE load was obtained by acceleration values in 3-dimensional directions, as defined in the KEPIC MFA, as shown in Table 2.  Figure 7 shows a contour plot of the applied stresses and deformations, and the results are listed in Table 3. A maximal stress of 135 MPa occurred at the contact area between the topside of the stem and the body under a load combination of the dead weight, the operation load under reverse pressure, and the SSE load. The safety factor that described the structural capacity of the butterfly valve was 1.7 in consideration of the allowable stress of the material that was used for the body, Gr. WCB, with a yield strength of 235 MPa.

### 4.2. Dynamic Analysis

#### 4.2.1. The Procedure for Response Spectrum Analysis

**Figure 8:**
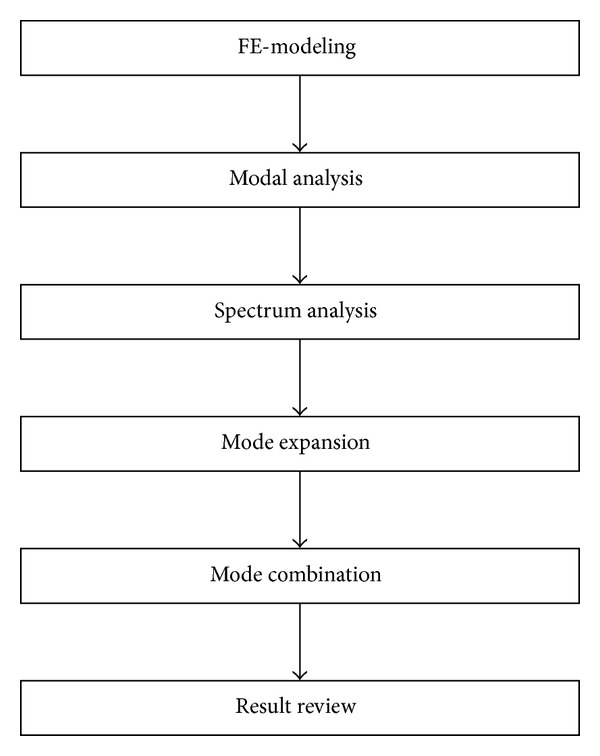
Flow chart for response spectrum analysis based on dynamic analysis.

If the butterfly valve is installed in a pipeline system, its natural frequency could be lower than 33 Hz, and a resonance failure might be expected. In this case, dynamic analysis should be performed, so that the pipeline system, including the butterfly valve, could show a flexible dynamic motion. The procedure for response spectrum analysis is provided in the KEPIC END [16]. This procedure is based on dynamic analysis, as mentioned in Section 2.2.2.  Figure 8 shows a flow chart for the response spectrum analysis.

#### 4.2.2. Characteristics of Dynamic Behavior

**Figure 9:**
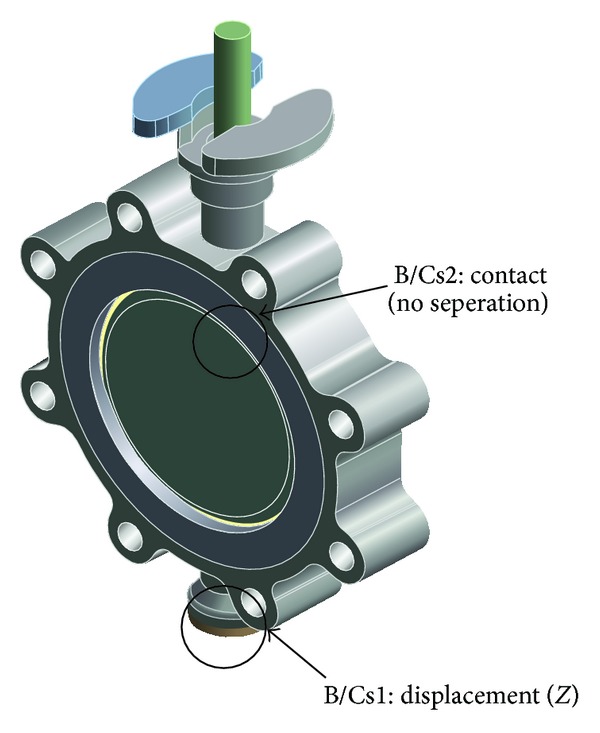
Boundary conditions for modal analysis based on dynamic analysis.

**Figure 10:**
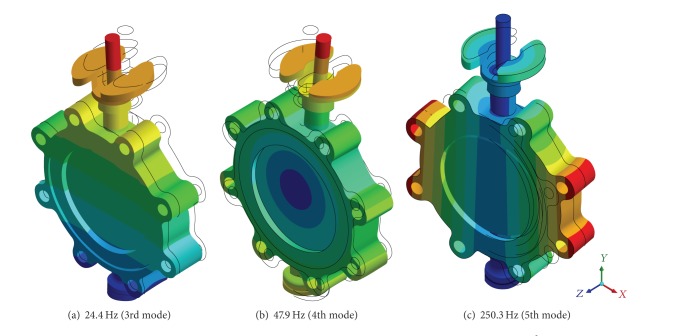
Frequencies at each mode obtained by modal analysis based on dynamic analysis.

For the response spectrum analysis, the natural frequencies of the butterfly valve were obtained by the modal analysis for a full-scale model of a pipeline system, which included this valve. The present study used a simple method to simulate the dynamic behaviors of the full-scale model. This was implemented by modification of the boundary conditions, in which all of the constraints at the valve body were released except for a displacement in the *z*-direction.  Figure 9 shows a schematic diagram of the boundary conditions for the modal analysis that was based on the dynamic analysis.  Figure 10 shows the natural frequencies at each mode. The 1st and 2nd modes did not occur due to its free-body motion. Meanwhile, the natural frequencies at the 3rd, 4th, and 5th modes were calculated as 24.4, 47.9, and 250 Hz, respectively. The natural frequency at the 3rd mode was lower than 33 Hz, so that the pipeline system including the butterfly valve would be considered flexible and could be affected by the resonance in the dominant frequency range of an earthquake.

#### 4.2.3. Structural Safety Assessment by Response Spectrum Analysis

**Figure 11:**
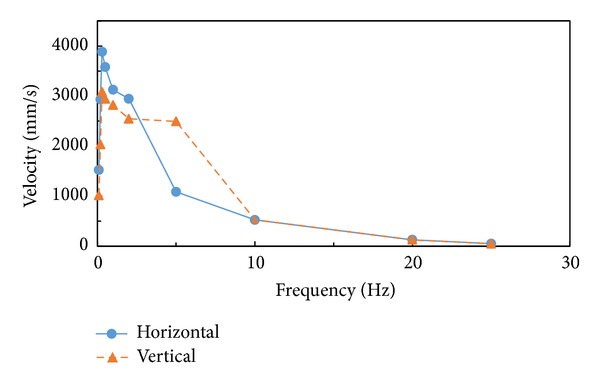
Design response spectrum considering a 2% damping ratio in the horizontal and vertical directions, as stipulated in the KEPIC MFA.

**Figure 12:**
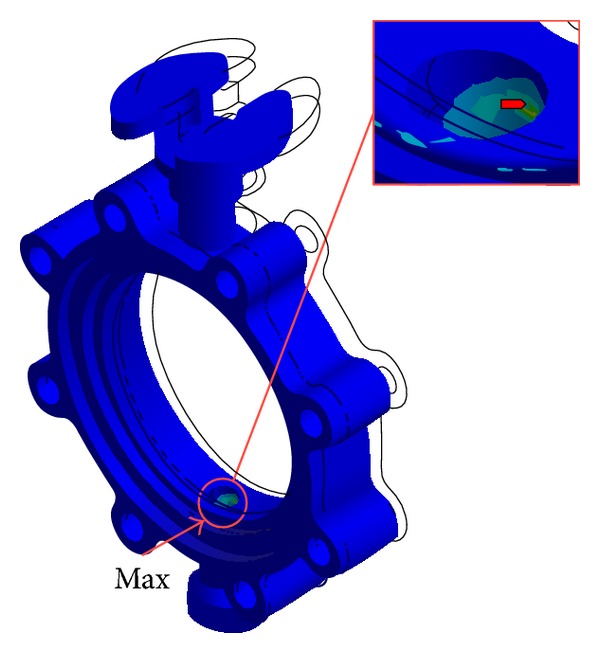
Results of structural analysis obtained by dynamic analysis.

For the response spectrum analysis, the responses of stress obtained from each modal response at all significant modes were combined according to the CQC method based on the use of cross-modal coefficients, as shown in (3). The structural analysis that was used to estimate the responses of stress took into account all three load conditions suggested in the KEPIC MFA, that is, RRS (required response spectrum), DRS (design response spectrum), and RIM (required input motion). In general, the DRS is recommended if the RRS and the RIM are not available. In the present study, the load condition regulated in the KEPIC MFA, as shown in Figure 11, was granted to the DRS where a damping ratio of 2% in the horizontal and vertical directions was taken into account in a case where the valves would be applied to a nuclear power plant.  Figure 12 shows the results of the structural analysis, in which the maximal stress of 183 MPa occurred at the contact area between the bottom layer of the stem and the body. The characteristics of dynamic behavior were similar to that for the 3rd mode of the modal analysis, as shown in Figure 10. The structural safety factor was 1.3. Although this result is lower than that in the case of static analysis, the structural safety of the butterfly valve met the requirements of the KEPIC MFA.

## 5. Conclusions

In this study, the structural safety analysis of a 200A butterfly valve for use in a nuclear power plant was performed in static and dynamic ways according to the KEPIC MFA. The results are as follows.

1. Analytical and experimental modal tests were carried out, and their deviations were taken into account. The initial finite element model was modified to decrease the error range to less than 3%.
2. The static analysis provided a maximal stress of 135 MPa at the contact area between the topside of the stem and the body under a load combination of the dead weight, the operation load under reverse pressure, and the SSE load. The safety factor for the structural capacity of the butterfly valve was 1.7.
3. In the case of dynamic analysis, the maximal stress was 183 MPa, and the characteristics of dynamic behavior were similar to those for the 3rd mode of the modal analysis. The structural safety factor was 1.3. These values were under the allowable strength for the materials used in the manufacture of the butterfly valve, and, therefore, its structural safety met the requirements of the KEPIC MFA.

The presented findings could be applicable as an index of the structural safety of the butterfly valve based on the seismic qualification in a nuclear power plant. To verify the fatigue requirements according to ASME Section III, the frequency of occurrence based on operating histories should be taken into account, and the fatigue analysis is currently being carried out. These results will be presented elsewhere in the near future.

## Figures and Tables

**Table 1:** Comparison of natural frequencies obtained using initial and modified FE-models.

	1st mode	2nd mode	3rd mode
Natural frequency obtained from the experimental modal test (Hz)	69.3	72.2	219.7
Natural frequency obtained by using the initial FE-model (Hz)	92.7 (25%)	96.4 (25%)	236.6 (7.7%)
Natural frequency obtained by using the modified FE-model (Hz)	68.9 (0.5%)	72.3 (0.1%)	226.6 (3%)

() means % errors (abs.) to natural frequencies obtained from experimental modal test.

**Table 2:** Acceleration values of the SSE load according to the KEPIC MFA.

Horizontal (*x*-dir.)	Horizontal (*y*-dir.)	Vertical (*z*-dir.)
4.5 G	4.5 G	3.0 G

**Table 3:** Results of structural analysis based on static analysis for Grade D of the KEPIC MFA.

	Max. stress (MPa)	Safety factor
Normal pressure	57	4.1
Reverse pressure	135	1.7
